# Conjugation with RGD Peptides and Incorporation of Vascular Endothelial Growth Factor Are Equally Efficient for Biofunctionalization of Tissue-Engineered Vascular Grafts

**DOI:** 10.3390/ijms17111920

**Published:** 2016-11-16

**Authors:** Larisa V. Antonova, Alexander M. Seifalian, Anton G. Kutikhin, Victoria V. Sevostyanova, Vera G. Matveeva, Elena A. Velikanova, Andrey V. Mironov, Amin R. Shabaev, Tatiana V. Glushkova, Evgeniya A. Senokosova, Georgiy Yu. Vasyukov, Evgeniya O. Krivkina, Andrey Yu. Burago, Yuliya A. Kudryavtseva, Olga L. Barbarash, Leonid S. Barbarash

**Affiliations:** 1 Research Institute for Complex Issues of Cardiovascular Diseases, Sosnovy Boulevard 6, Kemerovo 650002, Russia; antonova.la@mail.ru (L.V.A.); sevostyanova.victoria@gmail.com (V.V.S.); matveeva_vg@mail.ru (V.G.M.); telella@mail.ru (E.A.V.); a.mir.80@mail.ru (A.V.M.); bio.tvg@mail.ru (T.V.G.); sergeewa.ew@yandex.ru (E.A.S.); v.georgiy@mail.ru (G.Y.V.); leonora92@mail.ru (E.O.K.); keu-73@mail.ru (A.Y.B.); jackie1970@mail.ru (Y.A.K.); olb61@mail.ru (O.L.B.); director@kemcardio.ru (L.S.B.); 2 Centre for Nanotechnology and Regenerative Medicine, UCL Division of Surgery and Interventional Science, University College London, UCL Medical School Building, 21 University Street, London WC1E 6AU, UK; a.seifalian@gmail.com; 3 NanoRegMed Ltd., 20-22 Wenlock Road, London N1 7GU, UK; 4 Kemerovo Cardiology Dispensary, Sosnovy Boulevard 6, Kemerovo 650002, Russia; reception@kemcardio.ru

**Keywords:** tissue engineering, vascular graft, RGD peptides, vascular endothelial growth factor, endothelialization

## Abstract

The blend of poly(3-hydroxybutyrate-*co*-3-hydroxyvalerate) (PHBV) and poly(ε-caprolactone) (PCL) has recently been considered promising for vascular tissue engineering. However, it was shown that PHBV/PCL grafts require biofunctionalization to achieve high primary patency rate. Here we compared immobilization of arginine–glycine–aspartic acid (RGD)-containing peptides and the incorporation of vascular endothelial growth factor (VEGF) as two widely established biofunctionalization approaches. Electrospun PHBV/PCL small-diameter grafts with either RGD peptides or VEGF, as well as unmodified grafts were implanted into rat abdominal aortas for 1, 3, 6, and 12 months following histological and immunofluorescence assessment. We detected CD31^+^/CD34^+^/vWF^+^ cells 1 and 3 months postimplantation at the luminal surface of PHBV/PCL/RGD and PHBV/PCL/VEGF, but not in unmodified grafts, with the further observation of CD31^+^CD34^−^vWF^+^ phenotype. These cells were considered as endothelial and produced a collagen-positive layer resembling a basement membrane. Detection of CD31^+^/CD34^+^ cells at the early stages with subsequent loss of CD34 indicated cell adhesion from the bloodstream. Therefore, either conjugation with RGD peptides or the incorporation of VEGF promoted the formation of a functional endothelial cell layer. Furthermore, both modifications increased primary patency rate three-fold. In conclusion, both of these biofunctionalization approaches can be considered as equally efficient for the modification of tissue-engineered vascular grafts.

## 1. Introduction

In accordance with the recent statistics, 17.3 million people died of cardiovascular disease (CVD) in 2013 [1], with a trend to increasing incidence at least until 2030 [2]. The vast majority of CVD-related deaths (>85%) are due to atherosclerosis [1] leading to ischemic stroke, ischemic heart disease, and peripheral artery disease [3]. Blood vessel replacement and bypass surgery are widely established as appropriate options for the treatment of severe atherosclerosis [4]. Both of them require a vascular conduit; i.e., an autologous great saphenous vein, radial artery, or internal mammary artery [5]. Nevertheless, the use of autografts has certain drawbacks, such as the inconvenience of harvesting and limited availability in patients with severe widespread atherosclerosis or in those who previously underwent bypass surgery [6]. Synthetic vascular grafts made of expanded poly(tetrafluoroethylene), poly(ethylene terephthalate), or polyurethane have been validated as appropriate conduits for medium and large caliber (>6 mm) arteries [5,7]. However, their performance in small caliber (≤6 mm) arteries are generally poor, with a patency rate of only 25% at 3 years postimplantation [5]. The reasons for this include intimal hyperplasia and thrombosis secondary to a low blood flow, lack of endothelialization, high shear, and compliance mismatch [7,8].

Vascular tissue engineering is considered as a promising approach for producing biocompatible and mechanically competent small caliber vascular substitutes [9]. Many types of natural and synthetic biodegradable polymers have been investigated and used to prepare tubular grafts for in situ vascular tissue engineering [7]. Natural polymers display excellent biocompatibility and biodegradability with non-toxic end products; however, they are highly immunogenic, have poor processability, and their mechanical properties are commonly insufficient for artificial vascular grafts [7,10,11]. On the other hand, synthetic polymers usually have low immunogenicity, excellent mechanical properties, and processability, but lack biocompatibility (e.g., cell recognition sites) [7]. To overcome these problems, we applied polymer blending, a widely established approach, to prepare a desirable bio-composite for the tissue-engineered vascular graft [5,7,10].

For the blending, we selected poly(ε-caprolactone) (PCL) and poly(3-hydroxybutyrate-*co*-3-hydroxyvalerate) (PHBV). PCL is a semi-crystalline aliphatic polyester synthesized via ring-opening polymerization of ε-caprolactone [7]. PCL is well known for its unique mechanical properties [11,12], slow biodegradation, low immunogenicity, and good drug permeability [7]. However, PCL has poor cell affinity due to its high hydrophobicity and lack of cell-binding signals; this significantly hinders endothelialization of the luminal surface [7]. PHBV is a highly crystalline aliphatic polyester produced by microorganisms as a storage compound [13]. As a natural biomaterial, it has no residual impurities, catalysts, or initiators [12], and possesses slow biodegradability [14] and high cytocompatibility [15]. Hence, we hypothesized that a blend of PHBV with PCL would be efficient for vascular tissue engineering purposes. In our initial in vivo study, a quarter of electrospun small-diameter PHBV/PCL grafts demonstrated spontaneous endothelialization in a rat abdominal aorta replacement model [16]; however, a primary patency rate of 25% was not considered to be sufficiently high for starting a preclinical trial on large animals.

A number of strategies have been implemented to endow the luminal surface of vascular grafts with the ability to adhere endothelial cells [7]. Most of them involve immobilizing cell adhesive proteins or bioactive peptides on the surface to enhance endothelial cell adhesion following rapid endothelialization [7]. In comparison with proteins, bioactive peptides have high stability and simple structure [7]. Furthermore, bioactive peptides can be immobilized onto material surfaces by either physical absorption or chemical reactions [7]. Incorporation of growth factors into polymer scaffolds during electrospinning is another popular strategy to promote cell adhesion and infiltration of the vascular grafts [7,10,11,17].

Therefore, we attempted to functionalize our grafts with either arginine–glycine–aspartic acid (RGD)-containing peptides or vascular endothelial growth factor (VEGF), both being widely established targets in vascular tissue engineering [7]. RGD is a tripeptide sequence widely distributed within extracellular matrix proteins (e.g., laminin, fibronectin, and von Willebrand factor/vWF) [18]. RGD is recognized as a ligand by integrins, receptors crucially important for cell adhesion, proliferation, survival, migration, and differentiation [19]. Hence, RGD peptides were proposed as a potential agent for improving polymer biocompatibility—particularly adhesive properties. Among the variety of available growth factors, we selected VEGF, the most powerful growth factor in the promotion of angiogenesis and vasculogenesis [20,21]. In addition to a plethora of other effects, VEGF induces the proliferation, survival, migration, and differentiation of endothelial cells [11,20,21].

The following studies by our group demonstrated that either conjugation with RGD peptides or the incorporation of VEGF improves biocompatibility, physico-mechanical properties, and enhances endothelialization of electrospun PHBV/PCL vascular grafts [22,23]. However, in vivo comparative assessment of the mentioned modifications in either short- or long-term periods is still lacking. Here we performed the implantation of unmodified grafts and grafts either conjugated with RGD peptides or with incorporated VEGF into rat abdominal aorta following histological and immunohistochemical examination at ascending time points.

## 2. Results

**Figure 1:**
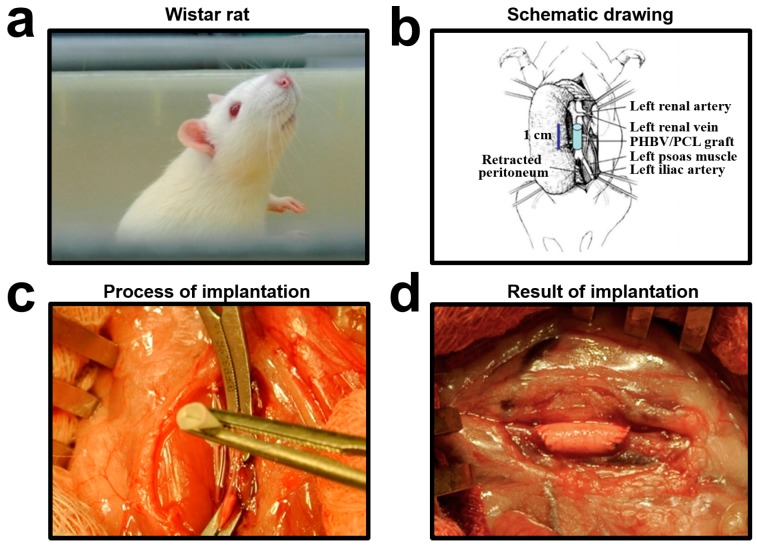
Implantation of the vascular grafts. (**a**) Wistar rat used for implantation; (**b**) schematic drawing of the surgery; (**c**) the process of implantation—aorta is temporarily clamped proximally and distally; (**d**) implanted graft, the anterior abdominal wall before the closure. PCL: poly(ε-caprolactone); PHBV: poly(3-hydroxybutyrate-*co*-3-hydroxyvalerate).

We first implanted RGD-, VEGF-, or non-treated PHBV/PCL grafts into abdominal aortas of Wistar rats (Figure 1). Rats were sacrificed 1, 3, 6, and 12 months postimplantation, and grafts were then collected for analysis.

**Figure 2:**
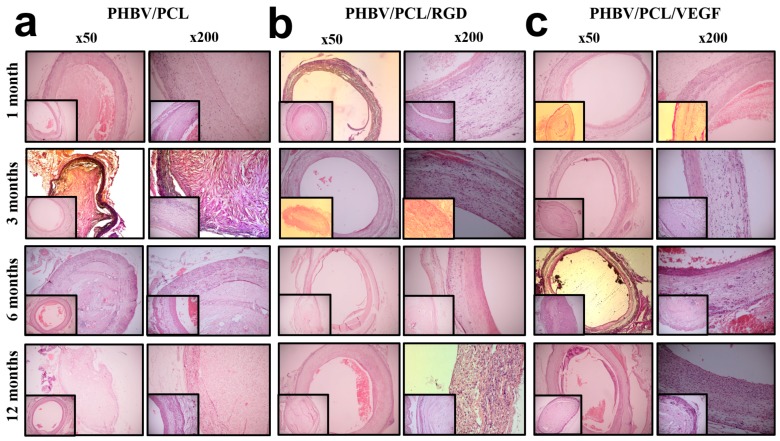
Hematoxylin and eosin (H & E) staining of the unmodified grafts and grafts with either conjugated arginine–glycine–aspartic acid (RGD) peptides or incorporated vascular endothelial growth factor (VEGF) at 1, 3, 6, and 12 months postimplantation; magnification: 50× and 200×. Representative images of: (**a**) poly(3-hydroxybutyrate-*co*-3-hydroxyvalerate)/poly(ε-caprolactone) (PHBV/PCL) grafts; (**b**) PHBV/PCL/RGD grafts; (**c**) PHBV/PCL/VEGF grafts. Images of corresponding patent PHBV/PCL along with occluded PHBV/PCL/RGD and PHBV/PCL/VEGF grafts are presented as bottom inserts.

It has been shown that transanastomosal ingrowth of cells is significantly more abundant in rats compared to human vasculature [24]. To avoid this bias, we assessed only midgraft area, and not anastomoses. Having performed histological examination of hematoxylin and eosin (H & E)-stained grafts, we observed a thrombotic occlusion within 75% (3/4) of unmodified PHBV/PCL grafts at all time points (Figure 2a). On the contrary, 75% (3/4) of the grafts modified with either RGD peptides (Figure 2b) or VEGF (Figure 2c) showed primary patency.

**Figure 3:**
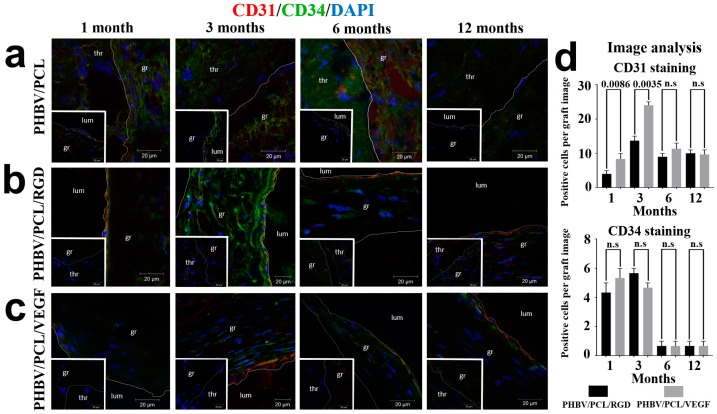
Combined CD31 (red), CD34 (green), and 4′,6-diamidino-2-phenylindole (DAPI, blue) staining. Representative images of: (**a**) PHBV/PCL grafts; (**b**) PHBV/PCL/RGD grafts; (**c**) PHBV/PCL/VEGF grafts. Thr is for thrombus, gr is for graft, lum is for lumen. Images of corresponding patent PHBV/PCL along with occluded PHBV/PCL/RGD and PHBV/PCL/VEGF grafts are presented as bottom inserts; (**d**) Quantitative image analysis, data are represented as mean with range, *p*-values are reported in a numerical manner, n.s. is for not significant, two-tailed Student’s *t*-test. Non-modified PHBV/PCL grafts were not included into analysis, since they were devoid of CD31^+^ and CD34^+^ cells.

Further, we performed a double immunostaining using well-recognized endothelial cell markers CD31 and CD34 [25,26] to assess endothelialization of the implanted grafts. Occluded unmodified grafts demonstrated aggregates of disorganized cells not characteristic of normalized vasculature (Figure 3a). In contrast, patent RGD- and VEGF-modified grafts showed CD31^+^/CD34^+^ cells at the luminal surface 1 and 3 months postimplantation, and CD31^+^/CD34^−^ cells 6 and 12 months postimplantation (Figure 3b,c). Strikingly, starting from month 3 of the experiment, cells at the luminal surface of patent grafts modified with either RGD peptides or VEGF exhibited an elongated phenotype characteristic of endothelial cells (Figure S1, bottom insert represents a positive control). When we compared two types of the modified grafts, differences in the number of CD31^+^ cells became insignificant in the sixth month; in addition, there were no differences in the number of CD34^+^ cells at all time points (Figure 3d).

**Figure 4:**
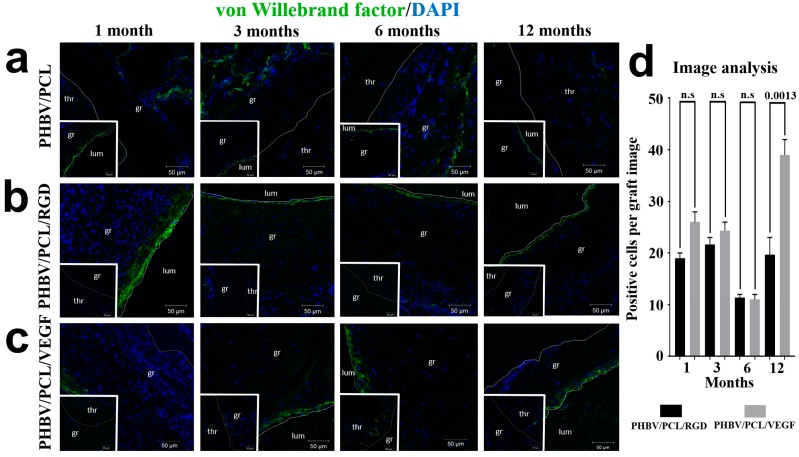
Combined von Willebrand factor (vWF, green) and DAPI (blue) staining. Representative images of: (**a**) PHBV/PCL grafts; (**b**) PHBV/PCL/RGD grafts; (**c**) PHBV/PCL/VEGF grafts. Thr is for thrombus, gr is for graft, lum is for lumen. Images of corresponding patent PHBV/PCL along with occluded PHBV/PCL/RGD and PHBV/PCL/VEGF grafts are presented as bottom inserts; (**d**) Quantitative image analysis, data are represented as mean with range, *p*-values are reported numerically, n.s. is for not significant, two-tailed Student’s *t*-test. Non-modified PHBV/PCL grafts were not included in the analysis, since their luminal surface was devoid of vWF^+^ cells, while the single positive cells within the graft wall were irrelevant for the assessment of endothelialization.

As an alternative marker of endothelial cells, we additionally stained grafts for the extracellular matrix protein von Willebrand factor (vWF), which establishes contacts between collagens, laminins, and endothelial cells [27]. We detected a layer of vWF^+^ cells at the luminal surface of patent grafts with either conjugated RGD peptides or incorporated VEGF, but not in occluded control grafts at all time points (Figure 4a–c). Differences in the number of vWF^+^ cells between two types of modified grafts became significant only to month 12 (Figure 4d). These results indicated the formation of an endothelial cell layer and improved endothelialization of RGD- and VEGF-treated grafts.

**Figure 5:**
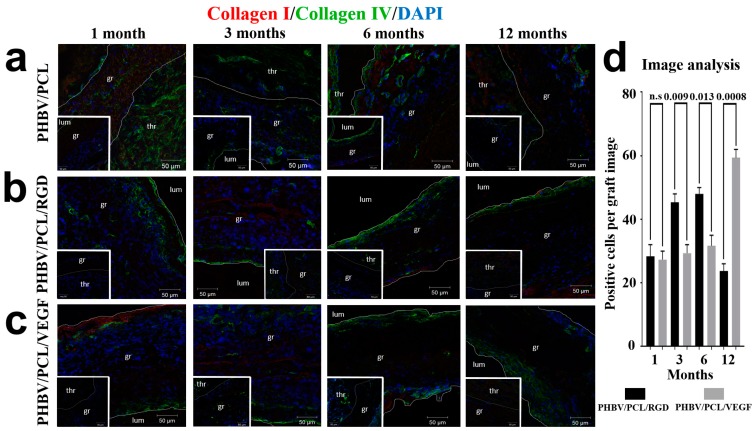
Combined collagen I (red), collagen IV (green), and DAPI (blue) staining. Representative images of: (**a**) PHBV/PCL grafts; (**b**) PHBV/PCL/RGD grafts; (**c**) PHBV/PCL/VEGF grafts. Thr is for thrombus, gr is for graft, lum is for lumen. Images of corresponding patent PHBV/PCL along with occluded PHBV/PCL/RGD and PHBV/PCL/VEGF grafts are presented as bottom inserts; (**d**) Quantitative image analysis, data are represented as mean with range, *p*-values are reported numerically, n.s. is for not significant, two-tailed Student’s *t*-test. Non-modified PHBV/PCL grafts were not included in the analysis, since their luminal surface was devoid of collagen I^+^ or collagen IV^+^ cells, while the single positive cells within the graft wall were irrelevant for the assessment of endothelialization.

Development of the basement membrane requires deposition of collagen chains I and IV [28]. To ask whether the observed endothelial cells are able to produce collagen for basement membrane development, we performed a double immunostaining for collagens I and IV. As expected, combined collagen I/IV staining identified a collagen-positive layer underneath the luminal surface of the patent grafts with either conjugated RGD peptides or incorporated VEGF at all the time points, but not in occluded unmodified grafts (Figure 5a–c). Differences in the number of collagen I/IV^+^ cells were ambiguous at distinct time points (Figure 5d). These data suggest the formation of basement membrane by endothelial cells upon RGD or VEGF treatment.

Of particular importance is that patent non-modified graft contained CD31^+^/CD34^+^ cells, a layer of vWF^+^ cells, and collagen IV (Figure 2a, Figure 3a, Figure 4a, and Figure 5a, bottom inserts). In contrast, non-patent RGD/VEGF-modified grafts were devoid of CD31, CD34, or vWF-positive cells, having aggregates of disorganized cells instead (Figure 2b,c; Figure 3b,c; Figure 4b,c; and Figure 5b,c; bottom inserts), as in occluded unmodified grafts. 

## 3. Discussion

We previously found that either conjugation with RGD peptides or incorporation of VEGF improve biophysical properties of electrospun PHBV/PCL small-diameter vascular grafts [22,23]. Modification with VEGF reduced mean pore area and mean fiber diameter in comparison with modification with RGD peptides [22,23]. Grafts with incorporated VEGF consisted mostly of nanofibers; on the contrary, those with RGD were formed mostly of microfibers [22,23]. As known, nanofibers enhance endothelial cell proliferation and adhesion as opposed to fibers with a larger diameter [29,30,31]; moreover, incorporation of VEGF allowed fabrication of the scaffolds with micro- to nanoscale topography similar to the natural extracellular matrix (ECM) [11,32,33]. Likewise, stress–strain curve, elasticity, and stiffness of the grafts modified with VEGF were closer to internal mammary artery in comparison with the RGD-modified grafts [22,23]. However, to the best of our knowledge, no studies compared in vivo efficiency of conjugation with RGD peptides and incorporation of VEGF in the same setting.

Here we conducted in vivo short- and long-term testing of electrospun PHBV/PCL small-diameter vascular grafts, including those with either conjugated RGD peptides or incorporated VEGF. Both of these biofunctionalization approaches improved endothelialization, promoted collagen production, and increased either short- or long-term primary patency three-fold compared to non-treated grafts. As a quarter of unmodified grafts were patent, we suggested that the PHBV/PCL blend itself is able to cause spontaneous endothelialization that can be significantly enhanced by either conjugation with RGD peptides or incorporation of VEGF. However, both non-modified and RGD/VEGF-modified grafts fail to develop features of a native vessel when early thrombosis occurs, possibly due to the lack of cell migration and perfusion.

Either RGD peptides or VEGF recruited CD31^+^/CD34^+^/vWF^+^ (i.e., “transitional” endothelial cells to the luminal surface) 1 and 3 months postimplantation, with further differentiation to CD31^+^/CD34^−^/vWF^+^ (i.e., mature endothelial cells) 6 and 12 months postimplantation. Both RGD peptide conjugation and VEGF incorporation resulted in the development of the vWF^+^ layer at all time points. Furthermore, endothelial cells synthesized collagen IV, forming a layer beneath the luminal surface that resembled a basement membrane. This can explain the increase in primary patency rate, since rapid endothelialization is well recognized as a key factor contributing to long-term patency [7,8]. However, neither visual examination nor quantitative image analysis identified differences in endothelialization pattern or patency between the grafts modified with either RGD peptides or VEGF. Despite enhanced biophysical properties provided by the incorporation of VEGF [22,23], we therefore considered these approaches as equally efficient. 

The use of either RGD peptides or VEGF to enhance the biocompatibility of polymers is not novel [34]. However, to the best of our knowledge, nobody has compared these two biofunctionalization approaches in the same setting (e.g., in a rat abdominal aorta replacement model). A limitation of our study is a low number of animals (*n* = 4) per each time point; nevertheless, we included four time points per graft type to better characterize the evolution of cellularity over time, which is one of the most interesting results of our approach. Another shortcoming, related to the rat model, is that transanastomosal ingrowth of cells is excessive in rats in comparison with humans [24]; thus, we assessed only midgraft areas. Detection of CD31^+^/CD34^+^ cells 1 and 3 months postimplantation with further change in phenotype to CD31^+^CD34^−^ also points to the cell adhesion from the bloodstream, but not on migration from anastomoses. 

We further plan to use a senescent sheep model for the evaluation of the small-diameter PHBV/PCL vascular grafts modified with either bioactive peptides or growth factors. The anatomic and hemodynamic conditions, as well as blood coagulation are similar to the human vasculature, while the long neck without any bifurcation provides easy access to the common carotid artery, which is a common site for implantation of 4–6 mm diameter vascular constructs [35,36,37]. Moreover, sheep represent a “worst-case model” due to the propensity for accelerated vascular calcification, allowing the assessment of the degenerative processes in a relatively short time period [37]. 

## 4. Materials and Methods

### 4.1. Graft Preparation

Electrospun vascular grafts were manufactured from PHBV/PCL (1:2, Sigma-Aldrich, St. Louis, MO, USA)/chloroform solution with and without VEGF, as in [23].

### 4.2. Polymer Amination–Activation

Grafts were processed as in [38,39] following air drying.

### 4.3. RGD Peptide Conjugation

Linker solution was prepared as in [40]. The synthesis of the RGD-containing biomolecule was performed as in [41]. Incubation in the linker solution and following treatment with the RGD solution (0.2 mg/mL) was carried out as in [22,39]. 

### 4.4. In Vivo Implantation

For the animal experiments, we used Wistar rats (male, 6-month-old, 400–450 g body weight, *n* = 48). The ethical committee of Research Institute for Complex Issues of Cardiovascular Diseases approved the study protocol (project No. 14-25-00050, 18 September 2014). Ethylene oxide-sterilized 10 mm length and 2 mm diameter PHBV/PCL, PHBV/PCL/RGD, and PHBV/PCL/VEGF (*n* = 16 per group) grafts were placed into abdominal aorta. Animal handling and surgery were performed as in [23]. One-fourth (*n* = 4) of rats in each of the groups was sacrificed 1, 3, 6, and 12 months postimplantation by an intraperitoneal injection of a sodium pentobarbital (100 mg/kg body weight). 

Equal parts of the explanted grafts and residual aortic tissue were either frozen at −140 °C or fixed in 10% formalin, as in [23]. 

### 4.5. Histological Examination

After being fixed with formalin, grafts were embedded in paraffin, further stained with hematoxylin and eosin, and finally examined by light microscopy (Axio Imager A1, Carl Zeiss, Cohen, Germany), as in [23].

### 4.6. Immunofluorescence Examination

Snap-frozen tissue samples were sectioned, treated, and stained as in [23], with additional staining by unconjugated mouse anti-collagen I (ab6308, Abcam, Cambridge, MA, USA) and rabbit anti-collagen IV (ab6586, Abcam). Native rat aorta and bovine serum albumin solution were used as a positive (Figure S2a) and negative (Figure S2b) control, respectively. Slides were examined by confocal laser scanning microscopy using LSM 700 (Carl Zeiss), as in [23]. Quantitative image analysis was conducted utilizing ImageJ software (National Institutes of Health, Bethesda, MD, USA). For the correct quantification, we first normalized each signal individually to the corresponding DAPI (4′,6-diamidino-2-phenylindole) signal of each vision field. Furthermore, we manually counted the number of positive cells in each vision field (three vision fields per staining; four stainings per time point) following quantitative image analysis for all the time points.

### 4.7. Scanning Electron Microscopy

Segments of explanted grafts (*n* = 4 per time point) were fixed with 2.5% glutaraldehyde (Sigma-Aldrich) during 24 h and then postfixed with 1% osmium tetroxide (Serva Electrophoresis) during 2 h at room temperature following dehydration in a graded ethanol series (from 30% to 100%) and further drying at 37 °С. Visualization was performed on a scanning electron microscope (Hitachi S-3400N, Hitachi, Chiyoda, Tokyo, Japan) in a low vacuum mode at 30 kV accelerating voltage, without any coating. Native rat aorta was used as a positive control.

### 4.8. Statistical Analysis

Statistical analysis was carried out utilizing GraphPad Prism (GraphPad Software). A sampling distribution was evaluated by Kolmogorov–Smirnov test and a D’Agostino–Pearson test. Descriptive data were represented by the mean with range. Two-tailed Student’s *t*-test was applied to compare independent groups, with further adjustment for multiple comparisons using Tukey’s post hoc test if needed. *p*-values ≤0.05 were defined as significant.

## Supplementary Materials

Supplementary materials can be found at www.mdpi.com/1422-0067/17/11/1920/s1.
