# Supplementary material for: Conjugation with RGD Peptides and Incorporation of Vascular Endothelial Growth Factor Are Equally Efficient for Biofunctionalization of Tissue-Engineered Vascular Grafts
Source: Int J Mol Sci. 2016 Nov 16;17(11):1920. doi: 10.3390/ijms17111920 (PMC5133917; doi:10.3390/ijms17111920)
Supplement: Supplementary file 1 [file ijms-17-01920-s001.pdf]

Supplementary Materials: Conjugation with RGD Peptides and Incorporation of Vascular Endothelial Growth Factor Are Equally Efficient for Biofunctionalization of Tissue-Engineered Vascular Grafts

Larisa V. Antonova, Alexander M. Seifalian, Anton G. Kutikhin, Victoria V. Sevostyanova, Vera G. Matveeva, Elena A. Velikanova, Andrey V. Mironov, Amin R. Shabaev, Tatiana V. Glushkova, Evgeniya A. Senokosova, Georgiy Yu. Vasyukov, Evgeniya O. Krivkina, Andrey Yu. Burago, Yuliya A. Kudryavtseva, Olga L. Barbarash and Leonid S. Barbarash

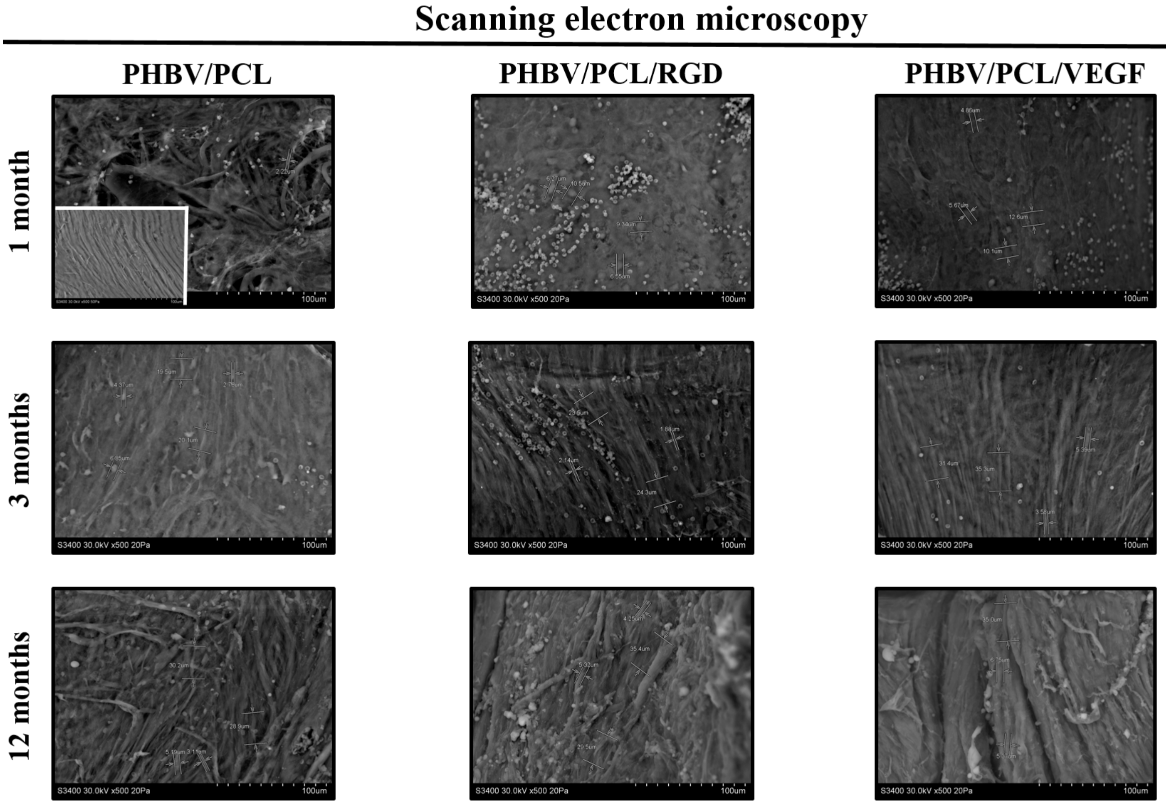

Figure S1. Scanning electron microscopy of the implanted grafts; bottom insert represents a positive control. PCL: poly(ε-caprolactone); PHBV: poly(3-hydroxybutyrate-co-3-hydroxyvalerate); RGD: arginine–glycine–aspartic acid; VEGF: vascular endothelial growth factor.

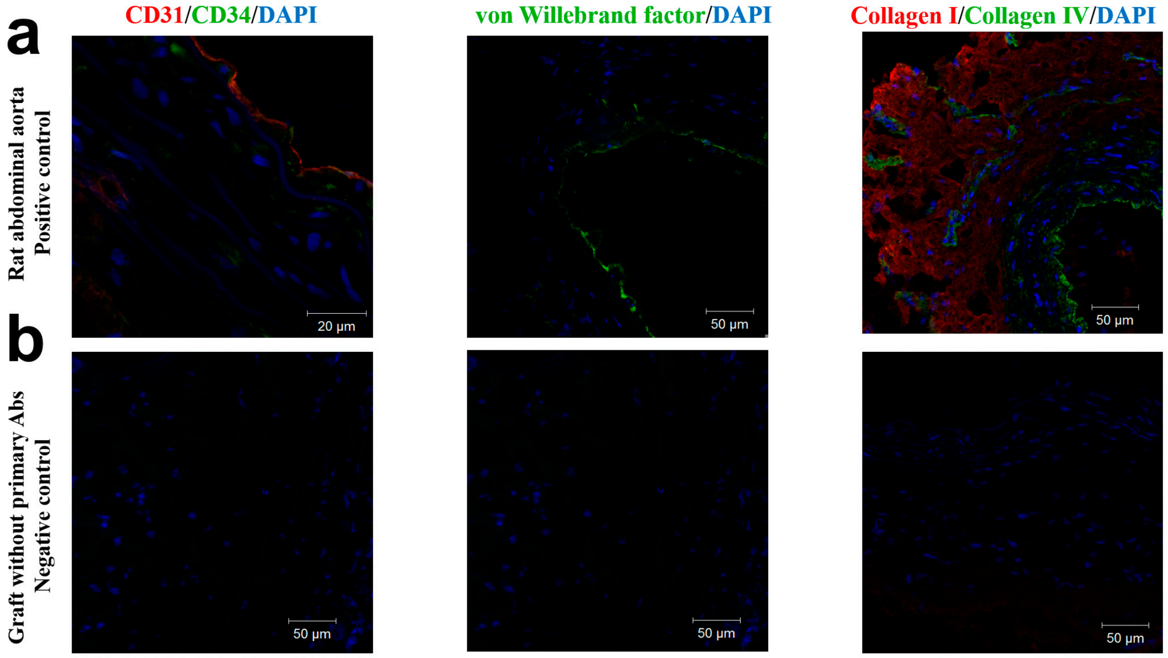

Figure S2. Positive and negative control for immunofluorescence staining. (**a**) rat abdominal aorta, positive control; (**b**) graft not incubated with primary antibodies, negative control. DAPI: 4′,6-diamidino-2-phenylindole.
